# A Semi-Supervised Speech Deception Detection Algorithm Combining Acoustic Statistical Features and Time-Frequency Two-Dimensional Features

**DOI:** 10.3390/brainsci13050725

**Published:** 2023-04-26

**Authors:** Hongliang Fu, Hang Yu, Xuemei Wang, Xiangying Lu, Chunhua Zhu

**Affiliations:** 1 Key Laboratory of Food Information Processing and Control, Ministry of Education, Henan University of Technology, Zhengzhou 450001, China; jackfu_zz@163.com (H.F.); yhang202303@163.com (H.Y.); xiangying1127@163.com (X.L.); zhuchunhua@haut.edu.cn (C.Z.); 2 Henan Engineering Laboratory of Grain IOT Technology, Henan University of Technology, Zhengzhou 450001, China

**Keywords:** deception detection, hybrid network, semi-supervised, feature fusion, consistency regularization

## Abstract

Human lying is influenced by cognitive neural mechanisms in the brain, and conducting research on lie detection in speech can help to reveal the cognitive mechanisms of the human brain. Inappropriate deception detection features can easily lead to dimension disaster and make the generalization ability of the widely used semi-supervised speech deception detection model worse. Because of this, this paper proposes a semi-supervised speech deception detection algorithm combining acoustic statistical features and time-frequency two-dimensional features. Firstly, a hybrid semi-supervised neural network based on a semi-supervised autoencoder network (AE) and a mean-teacher network is established. Secondly, the static artificial statistical features are input into the semi-supervised AE to extract more robust advanced features, and the three-dimensional (3D) mel-spectrum features are input into the mean-teacher network to obtain features rich in time-frequency two-dimensional information. Finally, a consistency regularization method is introduced after feature fusion, effectively reducing the occurrence of over-fitting and improving the generalization ability of the model. This paper carries out experiments on the self-built corpus for deception detection. The experimental results show that the highest recognition accuracy of the algorithm proposed in this paper is 68.62% which is 1.2% higher than the baseline system and effectively improves the detection accuracy.

## 1. Introduction

Lying is a common phenomenon in life [1] and is considered to be a high-level executive control behavior that causes activity in the amygdala, insula, and prefrontal regions of the brain [2], which in turn leads to changes in speech parameters such as frequency during speaking. There has been an attempt to improve the recognition rate of lie detection with advanced techniques. Deception detection techniques have been applied to criminal investigations [3], psychotherapy [4,5], children’s education [6], and national security [7] with some success. Traditional deception detection methods require contact with the human body, which may bring psychological burdens and interfere with the results of deception detection [8]. Aldert [9] also pointed out that the application of medical devices to collect physiological and brain signals may make these signals invasive and inconvenient to use, while speech signals can produce better results. Compared with traditional deception detection methods, speech deception detection methods have the advantages of easy access to data, absence of time and space constraints, and high concealment. Therefore, deception detection using speech has a strong theoretical and practical restudy value for the study of cognitive brain science [10].

Early relevant studies have confirmed that some acoustic features in speech are related to deception [11]. Ekman et al. [12] collected and analyzed the subjects’ impressions of some TV clips, and found that the fundamental frequency part of lies was higher than that of truth. Lying and stress are always related. Kaliappan and Hansen et al. [13,14] found that some acoustic parameters related to lying, such as resonance peak, Bark energy characteristics, and MFCC, changed with the alteration of pressure level. DePaulo et al. [15] meta-analyzed 158 features proposed by previous polygraph research work and selected 23 speech and speech-related features with significant expressions. The study found that lies showed less detailed expressions, repetitive utterances, more content, shorter expression lengths, and incoherent speech utterances compared to the truth. The research team of Purdue University in the United States used the amplitude modulation model and frequency modulation model to conduct speech deception detection research, proving that Teager energy-related features could distinguish truth from lie [16]. In addition, some relevant scholars considered combining multiple features for deception detection. Researchers at Columbia University considered combining acoustic features, prosodic features, and lexical features for research on lie detection in speech [17]. In 2013, Kirchhuebel et al. used the acoustic and temporal features of speech to study the effects of different conversation modes on deception detection from three aspects: emotional arousal/stress [18], cognitive load, and ultra control. Some scholars classify acoustic features into prosody features and spectral-based correlation analysis features [19]. Speech prosody refers to the vocal modulations that accompany speech and comprises variations in fundamental frequency, duration, and energy. In recent years, speech prosody has been recognized in several disciplines, including psycholinguistics, as a bridge between speech acts and mental disorders [20], and therefore has great research value in revealing the brain mechanisms behind speech communication. Spectral-based features can reflect the connection between speech tract shape and speech behavior [21]. The cochlea of the human ear is the key to forming hearing, which can convert speech signals into neural pulses and send them to the auditory area of the brain, generating hearing. The basilar membrane of the cochlea is equivalent to a nonlinear filter bank, and its movement frequencies are converted into nerve impulses by outer hair cells and inner hair cells. The mel-frequency cepstrum coefficient (MFCC) [22] is a feature parameter discovered based on this auditory mechanism, which is in nonlinear correspondence with frequency and has been widely used in the fields of speech emotion recognition and deception detection. Research has shown that early extraction and analysis of acoustic parameters affect the differentiation of early ERP responses, while stimuli caused by acoustic characteristics in the early stages can affect brain cognition in the later stages [23]. The nervous system encodes these evolving acoustic parameters to obtain a clear representation of different speech patterns, further enabling the brain to clearly distinguish between lies and truth. With the development of deep learning technology, researchers extracted deep features through deep neural networks and applied them to speech deception detection research. Xie et al. [24] combined spectral features that exploit the orthogonality and translational invariance of Hu moments with deep learning methods and used deep confidence networks for their experiments, achieving extremely high recognition results. Liang et al. [25] extracted speech-depth features using convolutional long and short-time memory networks, and achieved good recognition results on the self-built deception detection database.

Although the above scholars have made many achievements in the field of deception detection, the data-driven deep neural network is extremely dependent on large-scale labeled high-quality speech data, and the problem of insufficient data has become a key problem restricting the development of the field of voice-based lie detection [26]. The supervised model is the most common machine learning model, which is widely used in the field of speech deception detection and has achieved high recognition accuracy. When the amount of labeled data is insufficient, the improvement in lie detection accuracy by supervised models can appear to be inadequate. Unsupervised models, which can discover the intrinsic structure of data and are often used for data mining, may be particularly useful in cases where labeled data is not available or when there is a need to identify new patterns in speech. Due to the limitation of data volume, the application of unsupervised models in the field of lie detection in speech has yet to be further investigated. Semi-supervised learning is a learning method that combines supervised and unsupervised learning. Semi-supervised models learn the local features of a small amount of labeled data and the overall distribution of a large amount of unlabeled data to obtain acceptable or even better recognition results. Semi-supervised models offer a promising approach for lie detection in speech and other tasks. Tarvainen et al. [27] proposed a method of averaging the weights of the mean-teacher model, combined with the consistent regularization method, by adding perturbed data points to push the decision boundary to the appropriate location, improving the generalization of the model, and significantly improving the learning speed and classification accuracy of the network. Liu et al. [28] added a pseudo-label generation module under the framework of the classic domain confrontation network and reduced the impact of pseudo-label noise and the error rate of prediction results by introducing the mean-teacher model. In the field of speech deception detection, Fu H et al. [29] proposed a speech deception detection model based on a semi-supervised denoising autoencoder network (DAE), which achieved good results using only a small amount of labeled data. Due to the limitations of traditional acoustic features, the trained network representation ability is insufficient, and it is difficult to achieve high recognition accuracy. Su et al. [30] trained the BILSTM network and SVM models separately and further fused the classification results using a decision-level score fusion scheme to integrate all developed models. Fang et al. [31] proposed a speech deception detection strategy combining the semi-supervised method and the full-supervised method, and constructed a hybrid model combining semi-supervised DAE and fully supervised LSTM network, effectively improving the accuracy of semi-supervised speech deception detection. Although the above research has made some achievements, it ignores the exploration of the multifeature deception detection algorithm under a fully semi-supervised framework. Improper fusion of features can easily lead to poor generalization ability of the semi-supervised model.

Inspired by the feature fusion method and semi-supervised learning, this paper proposes a semi-supervised speech deception detection algorithm that integrates acoustic statistical features and time-frequency two-dimensional features to solve the problems in the research of speech deception detection, aiming to suppress the dimension disaster caused by multiclass feature fusion and obtain features with more favorable information in the semi-supervised learning environment. Firstly, the proposed algorithm employs a hybrid network composed of a semi-supervised AE network and a mean-teacher model network to extract the fusion features of deception detection, with the aid of the mean-teacher model to extract spectral features rich in time-frequency information, and applies a semi-supervised AE network to extract low-dimensional, high-level acoustic statistical features. Secondly, the consistency regularization method is introduced, and the dropout method is added to improve the generalization ability of the model and suppress the over-fitting phenomenon. Finally, the fusion features are input into the softmax classifier for classification, and the model is optimized by using a dynamically adjusted weighted sum of the cross-entropy loss of labeled data, the consistency regularization of unlabeled data, and the reconstruction loss of the AE network.

## 2. Materials and Methods

### 2.1. System Model

**Figure 1:**
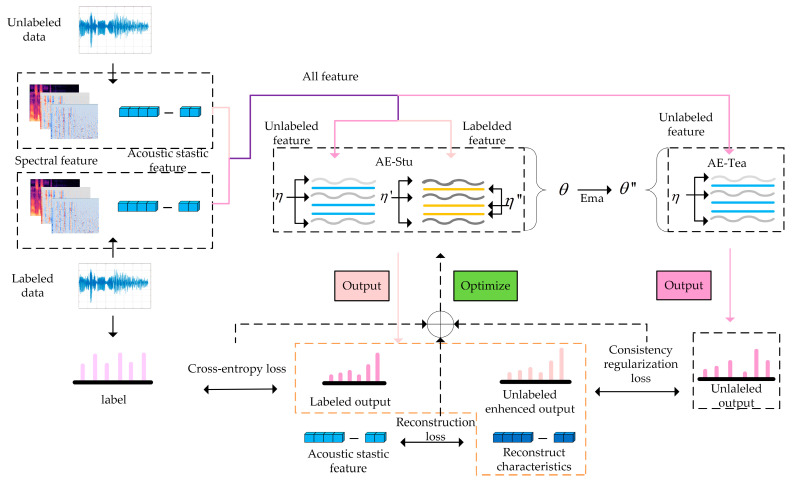
System model framework.

The proposed model framework is shown in Figure 1. The model applied the semi-supervised AE to obtain the depth acoustic statistical features, used the semi-supervised mean-teacher model based on the CNN network to extract the depth time-frequency two-dimensional features, and then employed the consistent regularization methods to constrain the fusion of the output features of the two semi-supervised networks to suppress the model over-fitting. Each module is described as follows.

### 2.2. Speech Feature Extraction

#### 2.2.1. Three-Dimensional Mel-Spectrum Feature

**Figure 2:**
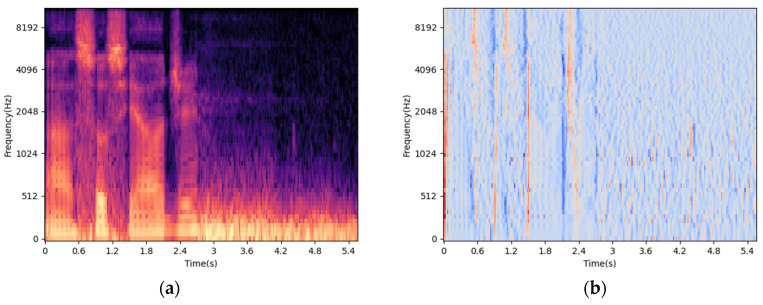

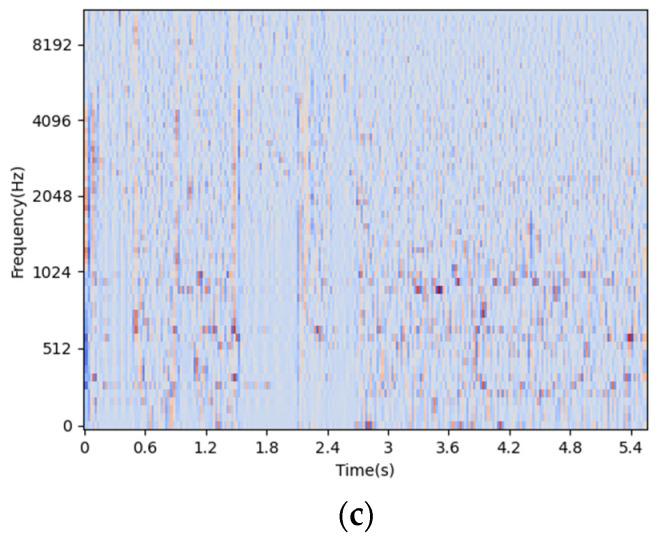
Components of 3D mel-spectrum. (**a**) shows the mel-spectrum, (**b**) shows the first-order difference feature, and (**c**) shows the second-order difference feature.

Lying can cause time-frequency changes in speech, and the mel spectrum has been proven to be rich in time-frequency features [32,33]. In this paper, 64 sets of filters, a 25 ms Hamming window, and a 10 ms overlap were used to obtain the features of the mel spectrum. By calculating the first-order difference and the second-order difference of the mel spectrum feature, and further supplementing the time-frequency information, the 3D mel-spectrum feature [34] was obtained, and its composition is shown in Figure 2. This article resized the 3D mel-spectrum feature to 256 × 256 × 3 as the input of the mean-teacher model, denoted by $X_{CNN}$.

#### 2.2.2. Acoustic Statistical Characteristics

Choosing the proper artificial statistical features is important for the effective learning of the model. Therefore, we employed the feature set [35] specified in the 2009 Sentiment Recognition Challenge in this paper. The feature set uses 16 low-level descriptors as well as 12 statistical functions, as shown in Table 1. The 16 low-level descriptors are, respectively, zero-crossing-rate (ZCR) from the time signal, root mean square (RMS) frame energy, pitch frequency (normalized to 500 Hz), harmonics-to-noise ratio (HNR) by autocorrelation function, and mel-frequency cepstral coefficients (MFCC) 1–12 in full accordance to HTK-based computation. To ensure the reproducibility of the experiments, we used opensmile software to extract these features from the feature set in speech. By calculating the low-level descriptors’ first-order difference and their 12 statistical functions, each speech could obtain 16 × 2 × 12 = 384 dimensional features as the input of the semi-supervised AE network, recorded as $X_{AE}$.

### 2.3. Semi-Supervised Hybrid Network Model

In this paper, we collected some labeled data $D_{L}={\left\{X_{}^{L},Y_{}^{L}\right\}}^{N_{L}}$, and a large number of unlabeled data $D_{U}={\left\{X_{}^{U}\right\}}^{N_{U}}$, where $Y_{}^{L}$ was the label, i=1,2,…,N, the total number of samples was $N=N_{L}+N_{U}$, $N_{L}$, and $N_{U}$, respectively, representing the total number of labeled data and unlabeled data. In this paper, the labeled data was recorded as $X^{L}=\{x_{AE}^{L},x_{CNN}^{L}\}$, and the unlabeled data was recorded as $X^{U}=\{x_{AE}^{U},x_{CNN}^{U}\}$. Among them, $x_{AE}^{L},x_{AE}^{U}$ were labeled input and unlabeled input of the semi-supervised AE network, with $X_{AE}=\{x_{AE}^{L},x_{AE}^{U}\}$; $x_{CNN}^{L},x_{CNN}^{U}$ were labeled input and unlabeled input of mean-teacher model, respectively, with $X_{CNN}=\{x_{CNN}^{L},x_{CNN}^{U}\}$.

#### 2.3.1. Mean-Teacher Model

**Figure 3:**
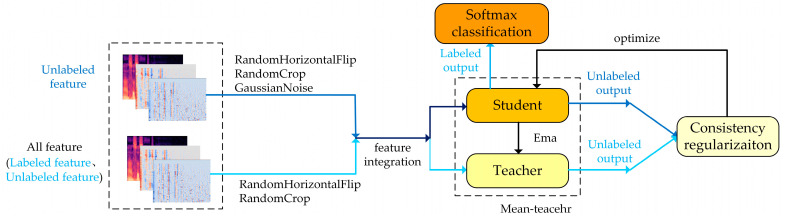
Structure of the mean-teacher model.

As described in Refs. [36,37], the mean-teacher model has achieved excellent recognition performance in the case of insufficient numbers of labels. The specific network structure is shown in Figure 3. The mean-teacher model consists of a student network and a teacher network, which are composed of the same convolutional neural network. Their structure is shown in Table 2. To increase the amount of data that could be processed, the weight relationship between each feature factor and the corresponding category in the speech data was established at a deeper level. In this paper, the spectral features were horizontally flipped and randomly clipped (the processing was recorded as η), and on that basis, unlabeled data was processed with noise enhancement (the processing was recorded as $\eta^{\prime }$), and the processed features were used as the input of the mean-teacher model. The output of the network is shown as follows:

(1) $$\begin{array}{l} Y_{Stu}^{L}=f_{CNN}(x_{CNN}^{L},Y_{}^{L},\eta ;\theta_{Stu})\\ Y_{Stu}^{U}=f_{CNN}(x_{CNN}^{U},\eta ,\eta^{\prime };\theta_{Stu})\\ Y_{Tea}^{U}=f_{CNN}(x_{CNN}^{U},\eta ;\theta_{Tea}) \end{array}$$

where $f_{CNN}$ refers to the process of feature extraction of the convolutional network. $Y_{Stu}^{L}$ refers to the output of labeled spectral data in the student network after processing η; $Y_{Stu}^{U}$ is the output of unlabeled spectral data on the student network after processing η and $\eta^{\prime }$. $Y_{Tea}^{U}$ is the output of unlabeled spectral data on the teacher network after processing η.

#### 2.3.2. Semi-Supervised AE Network

**Figure 4:**
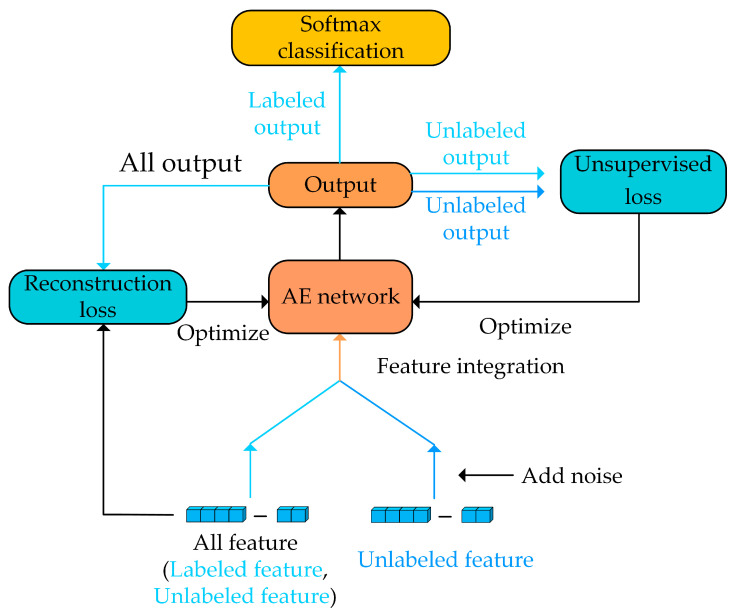
Structure of the AE model.

Refs. [38,39] show that the AE network can remove redundancy between features, fully dig the deep information between features, and obtain low-dimensional and high-level features. Therefore, a semi-supervised AE network was built in this paper and its parameter was $\theta_{AE}$. The network structure is shown in Figure 4, with its parameters can be seen in Table 3, and the extraction process is described as follows:

(2) $$\begin{array}{l} Y_{AE}^{L}=f_{AE}(x_{AE}^{L},Y_{}^{L};\theta_{AE})\\ Y_{AEN}^{U}=f_{AE}(x_{AE}^{U},\eta^{\prime \prime };\theta_{AE})\\ Y_{AE}^{U}=f_{AE}(x_{AE}^{U};\theta_{AE}) \end{array}$$

where $f_{AE}$ refers to the process of extracting features from the AE network, $\eta^{\prime \prime }$ refers to the noise enhancement processing of unlabeled features. $Y_{AE}^{L}$ refers to the output of labeled acoustic statistics in the AE network, $Y_{AEN}^{U}$ refers to the output of unlabeled acoustic statistics in the AE network after processing $\eta^{\prime \prime }$, and $Y_{AE}^{U}$ refers to the output of unlabeled acoustic statistics in the AE network. 

Reconstruction loss is the key to unsupervised learning by AE networks. By reducing the reconstruction error, it helps the models to extract high-level, high-quality features. The reconstruction loss of the AE network is shown as follows:

(3) $$L_{recon}=-\frac{1}{M}\sum_{i=1}^{M}\left([f_{RAE}(Y_{iAE})\cdot \log(Y_{iAE})+(1-f_{RAE}(Y_{iAE}))\log(1-Y_{iAE})]\right)$$

where M is the input number of each batch, $f_{RAE}$ refers to the process of reconstructing features from the AE network, $Y_{iAE}$ refers to the output of all features extracted by the AE network, and $f_{Rdae}(Y_{iAE})$ refers to the output obtained from manual statistical features after extraction and reconstruction by AE networks.

#### 2.3.3. The AE–MT Model with Consistent Regularization

**Figure 5:**
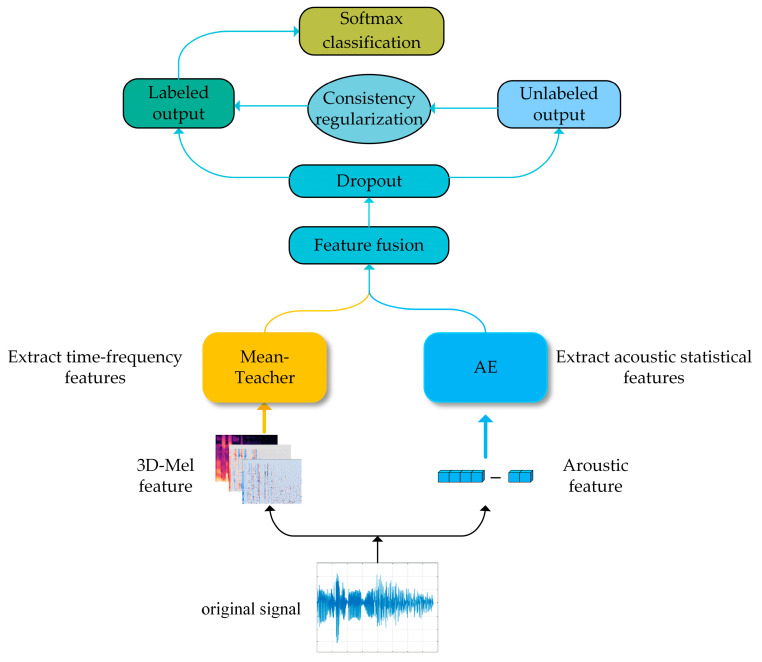
The flowchart of the AE–MT model with consistent regularization.

The semi-supervised AE–MT model constructed in this paper comprises two parts: the AE–Stu model and the AE–Tea model. The parameters of the AE–Stu model are expressed by $\theta =\{\theta_{AE},\theta_{CNN}\}$ and the parameters of the AE–Tea model are expressed by $\theta^{\prime }=\{\theta_{AE},{\theta_{CNN}}^{\prime }\}$. The AE–MT model flowchart is shown in Figure 5.

Taking advantage of the characteristic that the feature spaces of artificial statistical features and depth features are different, the AE–MT model fuses the two types of features to obtain high-level features with a strong ability of representation, including unlabeled enhanced output $Y_{AE\_StuN}^{U}=\{Y_{AEN}^{U},Y_{StuN}^{U}\}$, unlabeled output $Y_{AE\_Tea}^{U}=\{Y_{AE}^{U},Y_{Tea}^{U}\}$, and labeled output $Y_{AE\_Stu}^{L}=\{Y_{AE}^{L},Y_{Stu}^{L}\}$.

The supervised loss of the system model was represented by the cross-entropy loss function between the labeled output and the real label belonging to the AE–Stu model. Its equation is as follows:

(4) $$L_{ce}=-\frac{1}{M}\sum_{i=1}^{M}\left[Y_{i}^{L}\cdot \log(Y_{iAE\_Stu}^{L})+(1-Y_{i}^{L})\cdot \log(1-Y_{iAE\_Stu}^{L})\right]$$

**Figure 6:**
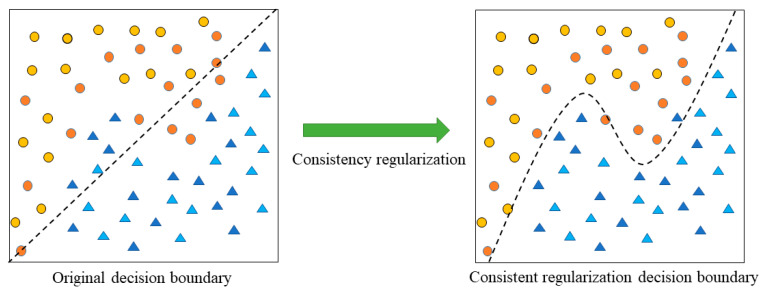
Consistency regularization optimization diagram. The circle (Ο) represents the truth feature, the triangle (Δ) represents the lie feature, yellow represents the undisturbed truth feature, orange represents the disturbed truth feature, light blue represents the undisturbed truth feature, and dark blue represents the disturbed lie feature.

The feature fusion method integrates different types of features to make up for the differences between features but also leads to the problem of dimension disaster, which will cause over-fitting and affect the classification ability of the model. Refs. [40,41,42] show that the consistency regularization method can utilize the potential information of unlabeled data and improve the generalization ability of the semi-supervised model. Meanwhile, to solve the problem that the perturbed fusion features are easily misclassified by the model, this algorithm introduces a consistency regularization method. As shown in Figure 6, although the original decision boundary is not enough to distinguish the perturbed features, the perturbed features are also correctly classified after optimization by the consistent regularization method.

In this paper, the loss of consistency regularization is defined as the expected distance between the unlabeled predicted output $Y_{AE\_StuN}^{U}$ of the AE–Stu model and the unlabeled predicted output $Y_{AE\_Tea}^{U}$ of the AE–Tea model.

(5) $$L_{consis}=\frac{1}{M}\sum_{i=1}^{M}\left[{\left||Y_{iAE\_StuN}^{U}-Y_{iAE\_Tea}^{U}|\right|}^{2}\right]$$

#### 2.3.4. Multiloss Model Optimization Model

In this paper, we optimized the AE–MT model using multiclass loss to improve its classification performance. The weighted sum of three types of losses was taken as the total loss of the AE–MT model, as shown below.

(6) $$L=L_{ce}+\omega \left(L_{consis}+a●L_{recon}\right)$$

where a is the weighting factor of the reconstruction loss function, and ω is the dynamically adjustable weighting factor of the sum of the consistent regularization loss and the reconstruction loss, which is generally constant.

The total loss was back propagated, the parameters $\theta_{}$ of the AE–Stu model were optimized and updated, and the parameters $\theta^{\prime }$ of the AE–Stu model were updated to those of the AE–Tea model through the exponential moving average method. The processing is shown below:

(7) $$\theta^{\prime \prime }=\alpha_{ema}\theta^{\prime }+\left(1-\alpha_{ema}\right)\theta$$

where $\alpha_{ema}$ represents the smoothing coefficient in the exponential moving average method, and $\theta^{\prime \prime }$ represents the updated parameters of the teacher network.

## 3. Experiment and Result Analysis

### 3.1. Dataset

**Figure 7:**
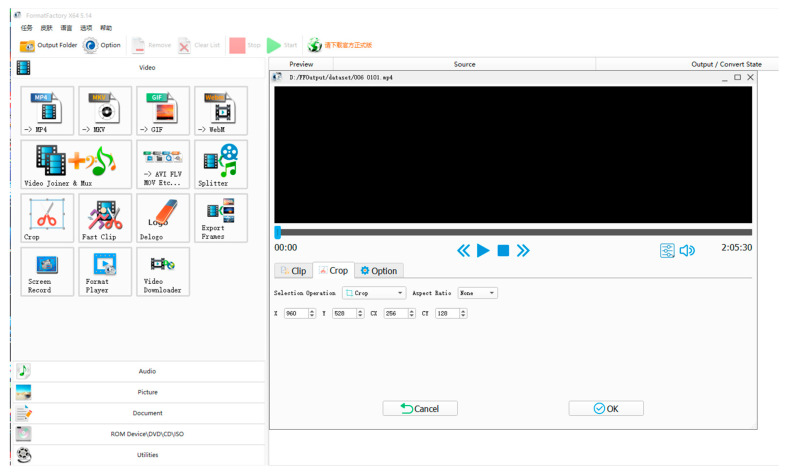
Interception of relevant video clips.

**Figure 8:**
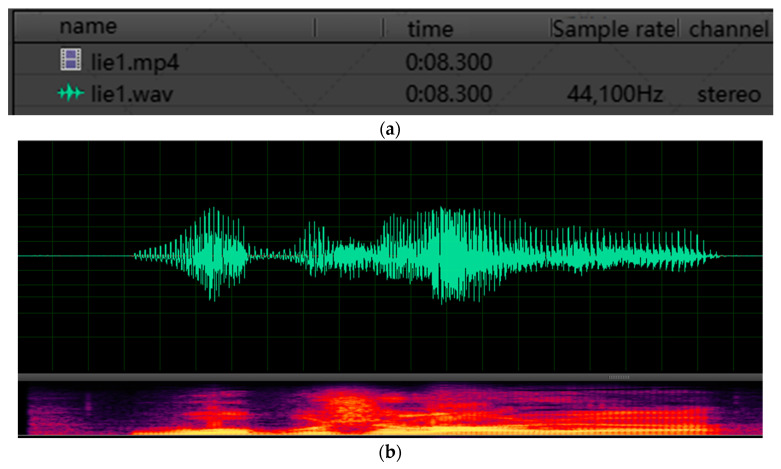
The extraction process of audio by Adobe Audition. (**a**) shows the separation of audio and video, and (**b**) shows the audio waveform and spectrogram.

In order to complement the existing corpus of deception detection and verify the effectiveness of the AE–MT model proposed in this paper, we built the H-Wolf corpus for speech deception detection experiments by referring to the construction process of the Idiap Wolf database [43], and the Killer database [29]. We collected about 70 h of the “Werewolves of Miller’s Hollow” competition video, which can be found on the internet, and screened video clips containing truth and lies according to the ID card of players and competition rules in each werewolf killing competition. Firstly, we used free audio and video editing tools, as shown in Figure 7, to intercept video clips related to truth or lie. We then used Adobe Audition, an audio processing software, to separate the audio from the video and display the audio waveform and spectrogram, which can be seen in Figure 8. Finally, we imported the audio clip using Adobe Audition software and modified its sample rate to 48,000 Hz, changing its bit depth to 16 bits. The number of players in each “Werewolves of Miller’s Hollow” competition is 12, and one player can participate in multiple competitions. After screening and statistics, the detailed number of participants is shown in Table 4. After multiple people tests, we retained clear and recognizable speech extracts, obtaining 1103 speech extracts (521 deception speech extracts). Then we divide them by 9:1 to obtain 992 training data and 111 test data. 

### 3.2. Experimental Configuration

In order to fully learn the feature information of the unlabeled data and reduce the impact of noise on the recognition results. we set *η* to 0.3 for enhancing unlabeled data. We used the small batch random gradient descent algorithm (SGD) for model training and set the learning rate to 0.0003. In this paper, the weight factor in Equation (4) is 0.5, and the cosine annealing attenuation method was used to adjust the learning rate so that the learning rate changed with the cycle.

We used accuracy and *f1_score* as classification evaluation criteria, accuracy as the main evaluation criterion for ablation experiments, and *f1_score* was used to further evaluate the performance of each module when the number of labels was 600. Their calculation method is shown in Formulas (8) and (9).

(8) $$accuracy=\frac{n_{correct}}{N_{total}}$$

(9) $$f1\_score=\frac{2\times TP}{2\times TP+FP+FN}$$

$n_{correct}$ represents the number of correctly predicted samples, $N_{total}$ represents the total number of samples. TP represents the positive samples with correct prediction, TN represents the negative samples with correct prediction, FP represents positive samples with prediction errors, FN represents negative samples with prediction errors.

To prevent data from over-fitting during training, we added dropout to the AE–MT model and set it to 0.8. All the experiments were carried out in the RTX 3080 and the 3.8 version of the python environment.

### 3.3. Results

#### 3.3.1. Ablation Study

To verify the classification performance of the fusion features of the proposed semi-supervised model compared with the single feature, we removed the mean-teacher network (base), the AE network, as well as the consistency regularization algorithm(CR), respectively, and then conducted speech deception detection experiments when the number of labeled data was set to 200, 400, 600, with other parameters unchanged. 

After a maximum of 100 epochs of iterative training, the experimental results are shown in Table 5 and Table 6.

As shown in Table 5, the accuracy of the mean-teacher model reached 61.35, 63.14, and 67.47% when the number of labeled data was 200, 400, and 600. From the results, the mean-teacher model made use of the potential information of unlabeled data and improved the accuracy of classification. The accuracy of the semi-supervised AE network in this paper achieved 63.0, 63.9, and 66.71% when the number of labeled data was 200, 400, and 600. It is noteworthy that the semi-supervised AE network attained the highest accuracy when the number of labeled data was 200. Because the AE network [44] is good at processing unsupervised data, even though the number of labeled data is fewer, the AE network can give better performance. However, when the number of labeled data is increased, the improvement in the semi-supervised AE networks’ performance is less than in the other model. According to the results of the AE + MT model in Table 5 and Table 6, it can be seen that simply combining the two models leads to dimensional disaster, causing an overfitting of the complex hybrid model, and reducing its classification performance. However, it should be noted that the AE + MT model combined with the consistent regularization method had better recognition performance than the other models. It was proved that the consistent regularization method could effectively solve the problem of overfitting of the model and improve the classification performance of the hybrid model. At the same time, the *f1_score* of the proposed model was also higher than the other models, confirming the effectiveness of the proposed method.

#### 3.3.2. Comparison to Other Algorithms

In addition, we compared the proposed algorithm with other semi-supervised methods. The comparison algorithm includes the semi-supervised AE model used in reference and the semi-supervised LSTM model using a pseudo-labeling algorithm. The differences between our algorithm and other algorithms are shown in Table 7 and Table 8.

As shown in Table 7, when the number of labels is 200, compared with SS-AE, and SS-LSTM, the values of the proposed method are improved by 8.87 and 7.92%. When the number of labels was 400, compared with SS-AE, and SS-LSTM, the accuracy of the proposed method increased by 10.51 and 6.77%. Compared with SS-AE and SS-LSTM, the accuracy of the proposed method increased by 13.24 and 10.7%, when the number of labels was 600. When the number of labels was different, the proposed algorithm always performed better than the other algorithms. As shown in Table 8, the *f1_score* of the proposed algorithm was much higher than the other algorithms, which further proves that the classification performance of the proposed model is much better than other models.

#### 3.3.3. Confusion Matrix

**Figure 9:**
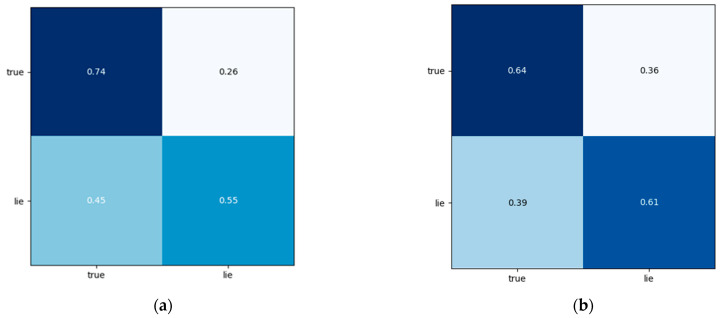

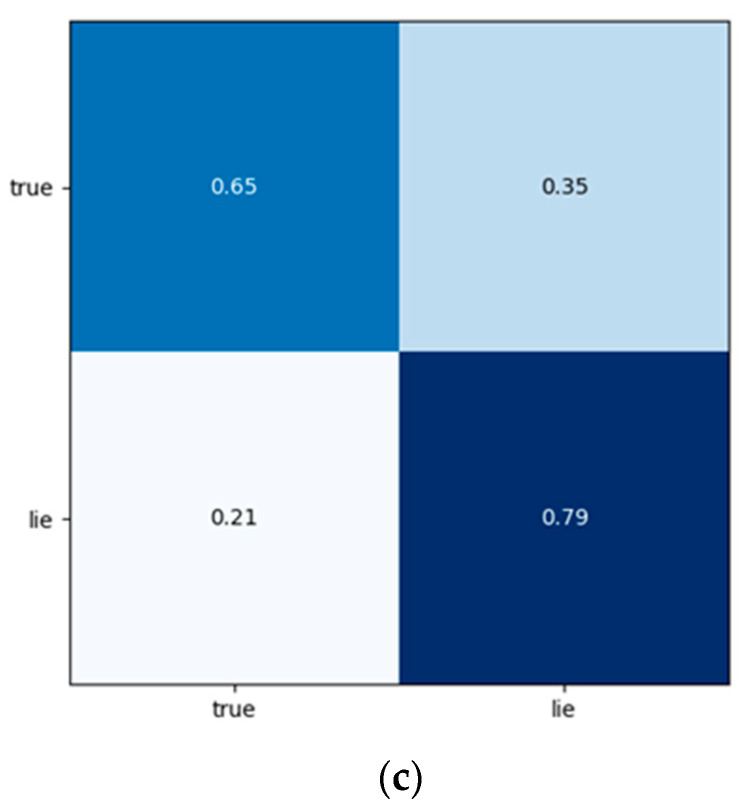
Confusion matrix of the different numbers of labels: (**a**) shows the confusion matrix of the model when the number of labels is 200, (**b**) shows the confusion matrix of the model when the number of labels is 400, and (**c**) shows the confusion matrix of the model when the number of labels is 600.

To further study the recognition accuracy, we introduced the confusion matrix to analyze the model. On the H-wolf dataset, we made the confusion matrix as shown in Figure 9. When the number of labeled data was 200, the recognition rate of truth and lies was 74 and 55%, respectively; when the number of labeled data was 400, the recognition rate of truth and deception was 64 and 61%, respectively; when the number of tags was 600, the recognition rate of truth and lies was 65 and 79%, respectively. Of these, the recognition rate of the truth was higher than 64%, and the accuracy rate of the lies was higher than 55%. With the increase in the number of labeled data, the recognition ability of the model to identify the lies was significantly improved.

## 4. Discussion

When people lie, they tend to use more complex language and take longer to respond to questions. This process is accompanied by changes in ERPs on the amygdala, insula, and prefrontal regions of the brain as well as changes in acoustic signature parameters associated with lying, with some studies demonstrating that these two changes are correlated [23]. Drawing on the work of Low et al. [47] and Pastoriza-Domínguez et al. [48] who used machine learning algorithms based on acoustic feature analysis for detecting major mental disorders, we focused, in this paper, on choosing the acoustic feature parameters associated with the act of lying and used the trained neural network model to detect subtle changes in the acoustic feature parameters under different speech patterns to discriminate between lies and truth. This can help us better understand how speech is processed in the brain and enable researchers to further investigate the brain’s cognitive neural mechanisms during the lying process. Our models can also be modified and applied to the assessment and diagnosis of speech prosody in mental disorders, in terms of the automatic classification of prosodic events for detection.

Due to the specific nature of the act of lying, it is difficult to insulate subjects from the effects of the equipment when collecting EEG signals and facial information related to lies, which can lead to biases between the data collected and the actual data. Moreover, in many cases, it is only after the act of lying has occurred that people’s brains become aware of the lie. As mentioned in the literature [49], the choice may have taken place before it was actually made. However, using speech signals alone for deception detection is not comprehensive; in some cases, EEG signals and facial information are more directly indicative of the true situation. Therefore, conducting multimodal lie detection research is meaningful [50], as it can comprehensively explore the neural mechanisms of the lying process from multiple perspectives.

## 5. Conclusions

In this work, we proposed a research framework for semi-supervised speech spoofing detection based on acoustic statistical features and time-frequency two-dimensional features. Unlike previous studies of semi-supervised speech spoofing detection algorithms based on a single feature and a single model, our proposed AE–MT model consists of two parallel components, namely, an AE network and an average teacher model, which deal with acoustic statistical features and time-frequency two-dimensional features, respectively. It is worth noting that applying feature fusion methods to the features extracted from the two networks can lead to high-level features with better representation. However, the feature fusion approach also increases the dimensionality, thus triggering a dimensionality catastrophe and exacerbating the overfitting of the model. Therefore, consistent regularization and dropout were also introduced in this paper to effectively improve the generalization ability of the model. Experiments showed that the AE–MT model could effectively mine feature information with good performance.

## Figures and Tables

**Table 1:** 2009 International Speech Emotion Recognition Challenge feature set.

Name	Specific Content
LLDs(16)	RMS, F0, ZCR, HNR, MFCC(1–12)
Statistical functions(12)	mean, stddev, kurtosis, skewness, max, min, maxposition, minposition, range, offset, slope, MSE

**Table 2:** The model parameters of student network and teacher network.

Network Layer	Output Shape
Input	256 × 256 × 3
3 × (Conv1 + BN + Relu)	256 × 256 × 32
Max-pooling	128 × 128 × 32
3 × (Conv2 + BN + Relu)	128 × 128 × 64
Max-pooling	64 × 64 × 64
Conv3 + BN + Relu	62 × 62 × 128
Conv4 + BN + Relu	62 × 62 × 64
Conv5 + BN + Relu	62 × 62 × 32
Sum and average	32
Full connection	8

**Table 3:** The model parameters of AE network.

Network	Network Layer	Number of Nerve Units
	Input	384
Encode network	Encode layer1 + BN + Elu	256
	Encode layer2 + BN + Elu	128
	Encode layer3 + BN + Elu	64
	Encode layer4 + BN + Elu + dropout	32
Decode network	Decode layer1 + BN + Elu	128
	Decode layer2 + BN + Elu	180
	Decode layer3 + BN + Elu	256
	Decode layer4 + BN + Elu + dropout	384
	Full connection	(32, 8)

**Table 4:** Number of Contestants.

Game Name	Male	Female	All
Werewolf	28	12	40

**Table 5:** The recognition accuracy of each model in the ablation experiment.

Database	Model	Labeled Examples		
		200	400	600
H-wolf	MT (base)	61.35%	63.14%	67.47%
	AE	63.0%	63.9%	66.71%
	AE + MT	59.62%	64.42%	67.31%
	AE + MT + CR (proposed)	62.5%	65.38%	68.62%

**Table 6:** The *f1_score* (%) of each model in the ablation experiment when the number of labels is 600.

Indicators	MT (Base)	AE	AE + MT	AE + MT + CR (Proposed)
*f1_score*	66.4%	65.3%	65.8%	69.6%

**Table 7:** Comparison of recognition accuracy (%) between the proposed algorithm and other algorithms.

Database	Model	Labeled Examples		
		200	400	600
H-wolf	SS-AE [45]	53.63%	54.87%	55.38%
	SS-LSTM [46]	54.58%	58.61%	57.92%
	Proposed	62.5%	65.58%	68.62%

**Table 8:** Comparison between *f1_score* (%) of the proposed algorithm and *f1_score* (%) of other algorithms when the number of labels is 600.

Indicators	SS-AE	SS-LSTM	Proposed
*f1_score*	45.5%	49.8%	69.6%

## Data Availability

Not applicable.
